# Biodegradation of 3-methyldiphenylether (MDE) by *Hydrogenophaga atypical* strain QY7-2 and cloning of the methy-oxidation gene *mdeABCD*

**DOI:** 10.1038/srep39270

**Published:** 2016-12-20

**Authors:** Qian Yang, Shu Cai, Shaowei Dong, Lulu Chen, Jifei Chen, Tianming Cai

**Affiliations:** 1 The College of Resources and Environmental Sciences, Nanjing Agricultural University, Nanjing 210095, People’s Republic of China; 2 Jiangsu Academy of Agriculture Science, Nanjing 210014, People’s Republic of China

## Abstract

3-Methyldiphenylether (MDE) is an important alkyl-substituted diphenyl ether compound that is widely used as an intermediate in the synthesis of pyrethroid insecticides. An efficient MDE-degrading strain QY7-2, identified as *Hydrogenophaga atypical*, was isolated from activated sludge for the first time. Strain QY7-2 can utilize MDE as the sole carbon and energy source and completely mineralize MDE. The degradation pathway of MDE was proposed in the strain through metabolites identification. A gene cluster involving in methy-oxidation of MDE was cloned from QY7-2 and expressed in *Escherichia coli* BL21 (DE3), and the products were purified by SDS-PAGE. The specific activities of the recombinant enzymes MdeAB, MdeC and MdeD were 113.8 ± 3.5, 274.5 ± 6.2 and 673.4 ± 8.7 nmol min^−1^ mg^−1^, respectively. These results provide the biochemical and genetic foundation of microbial degradation pathway of MDE and benefit the bioremediation of MDE-contaminated environments.

Diphenyl ethers are a class of aromatic compounds that contain two benzene rings connected by an oxygen atom and are important intermediates in organic synthesis to produce spices, synthetic resins and other chemical products^1^. Diphenyl ether compounds have been widely used as flame retardants in a vast array of consumer products since the 1970s, including building materials, electronics, furnishings, motor vehicles, plastics, polyurethane foams, and textiles^2^. Moreover, diphenyl ether compounds, such as alkyl-substituted diphenyl ethers, halogenated diphenyl ethers, phenoxy benzoic acid, pyrethroid and biphenyl ether herbicides, are also commonly used in industry. Long-term exposure to diphenyl ether compounds could cause neurodevelopmental toxicity and endocrine disruption in mammals^3^,^4^. Diphenyl ethers always have strong chemical and biological stability, increasing their potential harmfulness to the environment^5^.

Biodegradation involves the use of living microorganisms to degrade pollutants and is generally considered as a major factor determining the fate of pollutants in the environment^6^. Biodegradation is the most important natural attenuation process for many xenobiotic chemicals in the environment and is generally deemed a safe and effective way to remove persistent organic pollutants (POPs) from the environment^7^. Several diphenyl ethers degrading microorganisms have been studied, indicating that microbial metabolism plays a significant role in the dissipation of diphenyl ethers residues in the environment. Schmidt reported that a diphenyl ether-degrading bacterium *Sphingomonas sp*. SS3 follows a pathway in which oxygens are introduced at positions C1 and C2 to directly produce catechol and phenol as one-aromatic-ring metabolites^8^. *Sphingomonas* sp. PH-07 utilizes diphenyl ethers as the sole carbon resource for growth, resulting in phenol and 2-hydroxymuconic acid as the metabolites, after which a cleavage catalysed by dioxygenase gives rise to the formation 2-hydroxy maleic^9^. Sun *et al*. showed biosorption and biodegradation in the removal process of 2, 2, 4, 4-tetrabromodiphenyl ether (BDE-47) using the *Pseudomonas stutzeri* strain KS0013^10^.

3-Methyldiphenylether (MDE) is an important alkyl-substituted diphenyl ether compound that is widely used as an intermediate in the synthesis of pyrethroid insecticides. Schmidt, *et al*.^11^ isolated a MDE-degrading strain SS31 which was identified as *Sphingomonas. sp.* Metabolites identification indicated that SS31 could degrade MDE into nondegradable product 3-phenoxybenzoic acid. However, the researches about the related genes and enzymes in strain SS31 were not reported. The objective of this study was to screen a MDE-degrading strain and investigate the biodegradation mechanism of MDE. An efficient MDE-degrading strain was isolated from activated sludge and was identified as *Hydrogenophaga atypical*. In addition, three metabolites of MDE were identified, and the novel gene, *mdeABCD,* which encodes the enzymes and catalyses the methy-oxidation reaction, was cloned and functionally expressed in *E. coli* BL21 (DE3).

## Results and Discussion

### Isolation of the MDE-degrading strain and its biodegradation behaviour

#### Isolation and identification of QY7-2

A strain that could use MDE as the sole carbon source under aerobic conditions was successfully isolated and was designated as QY7-2. Strain QY7-2 was Gram-negative, aerobic, nonsporeforming, motile, rod-shaped. Colonies grown on LB agar at 30 °C were circular, slightly convex, smooth, pale yellow in colour, and had entire margins. The 16S rDNA gene sequence results indicated that strain QY7-2 formed a distinct lineage within the genus *Hydrogenophaga* (Fig. S1), showed 98.41% similarity to *Hydrogenophaga atypical* BSB41.8 T. The DNA G + C content of strain QY7-2 was 67.6 mol%, which fell within the range observed for other members of the genus *Hydrogenophaga*. Thus based on the results of morphological, physiological and biochemical characterization, phylogenetic analysis of the 16S rDNA gene sequence and DNA G + C content, strain QY7-2 was identified as *Hydrogenophaga atypical.*

#### Degradation characteristic study of QY7-2

**Figure 1:**
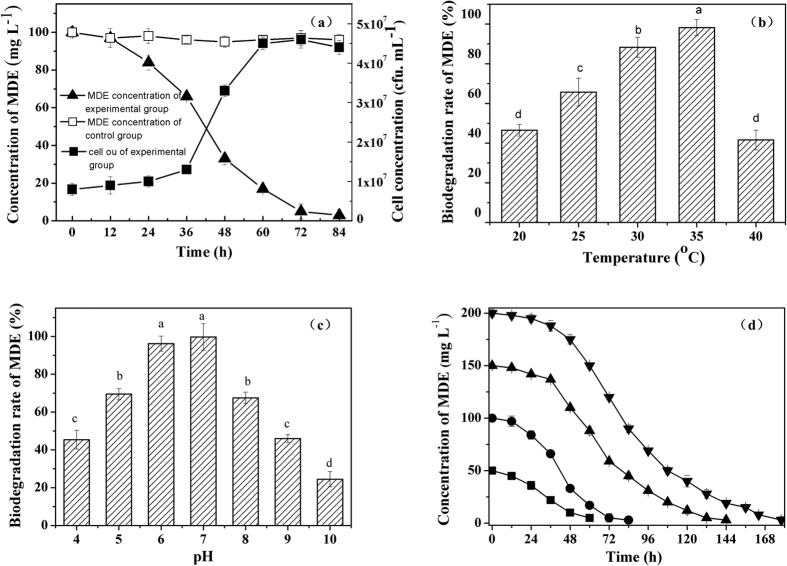
(**a**) MDE biodegradation profiles by strain QY7-2 and variations in cell concentration; (**b**, **c** and **d**) Influences of temperature, pH, and initial concentration on MDE degradation.

The degradation profiles of MDE (100 mg L^−1^) by strain QY7-2 are depicted in Fig. 1(a). Over 95% of the MDE was degraded by strain QY7-2 in 84 h, and the initial and final cell concentration values of QY7-2 were 8.0 × 10^6^ and 4.4 × 10^7^ cfu/mL. No significant change in the MDE concentration could be observed in the control group (inoculated with inactivated cells) indicating that the decrease of MDE concentration was due to microbial degradation process rather that adsorption mechanism. pH value and temperature are important factors that significantly influence the degradation of hazardous materials by microorganisms^12^,^13^. The degradation rates of 100 mg L^−1^ MDE after an 84 h incubation were 46.5%, 65.7%, 88.3%, 98.2%, and 41.6% of MDE at 20 °C, 25 °C, 30 °C, 35 °C, and 40 °C, respectively. The pH value also had a severe effect on the MDE degradation; strain QY7-2 degraded 45.4% (pH 4), 69.5% (pH 5), 96.2% (pH 6), 99.7% (pH 7), 67.5% (pH 8), 46.1% (pH 9) and 24.5% (pH 10) of the MDE compared with the optimal pH of 6–7. The effect of initial concentrations of MDE on biodegradation was shown in Fig. 1(d), when 50 mg L^−1^ was applied, there was no obvious lag period; when 200 mg L^−1^ MDE was applied, the degradation rate was lower at the beginning, but the MDE was still completely degraded within 180 h. With the increase of MDE concentration, the lag period prolonged. The lag phase might be caused by the toxicity of MDE to the bacterial cells.

**Figure 2:**
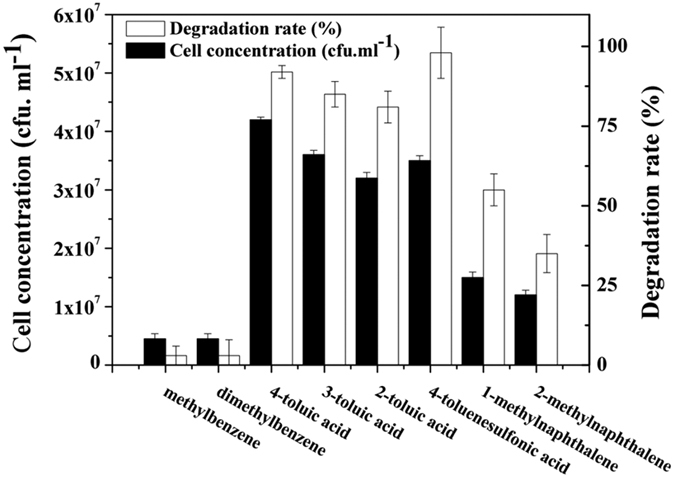
Degradation and utilization of various aromatic compounds by *Hydrogenophaga atypical* QY7-2 in MSM supplemented with 100 mg L^−1^ substrates. The cultures were incubated at 35 °C on a rotary shaker for 3d. The data were derived from three independent measurements, and the error bars indicate standard deviations.

The substrate spectrum that strain QY7-2 could degrade and utilize is shown in Fig. 2. Strain QY7-2 utilized over 80% of 4-toluic acid, 3-toluic acid, 2-toluic acid and 4-toluenesulfonic acid, but it could not utilize methylbenzene or dimethylbenzene. QY7-2 also degraded 1-methylnaphthalene and 2-methylnaphthalene, but the degradation was less than 60% over 3 d.

#### Kinetic study

**Figure 3:**
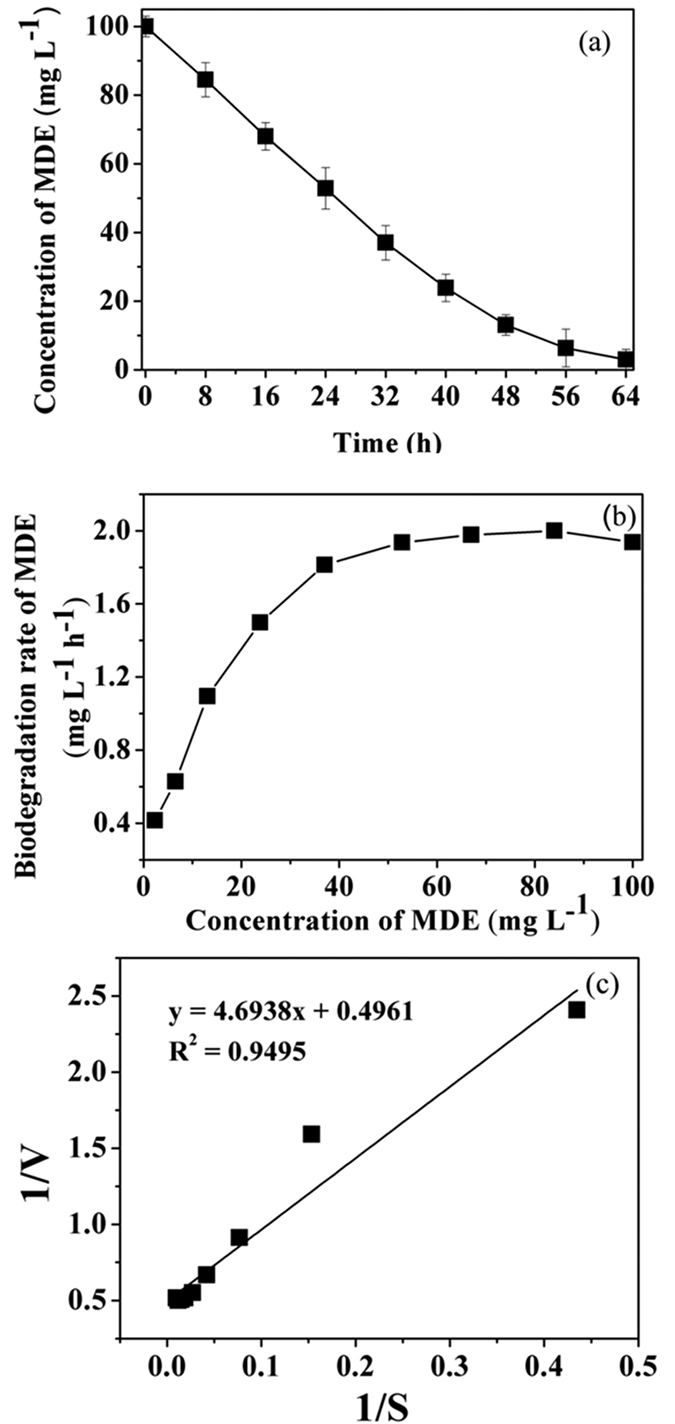
(**a**) Biodegradation behaviour of MDE by strain QY7-2 with an initial concentration of 100 mg L^−1^; (**b**) MDE degradation rates in different concentrations; (**c**) Application of Monod.

The biodegradation profiles of MDE, with an initial concentration of 100 mg L^−1^, are illustrated in Fig. 3a. Correspondingly, the specific degradation rates of MDE at different time intervals were 1.93, 2.01, 1.97, 1.94, 1.81, 1.50, 1.01, 0.63 and 0.41 mg L^−1^ h^−1^ (Fig. 3b). The degradation rates increased rapidly at the beginning of the reaction and finally stabilized.

By fitting the experimental data with Eq. (3), kinetic parameters were obtained (Fig. 3c): *V*_*max*_ = 2.01 mg L^−1^ h^−1^, *K*_s_ = 9.44 mg L^−1^. Correspondingly, the kinetic equation of the degradation process was shown in Eq. (1):


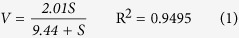


The monod equation indicated that the degradation rate was accelerated with the increase of substrate concentration. When the concentration of MDE was far less than 9.44 mg L^−1^, the degradation rate is proportional to the concentration of substrate; when the concentration of MDE was far more than 9.44 mg L^−1^, the degradation rate was close to *V*_*max*_ (2.01 mg L^−1^ h^−1^) and keeping nearly constant.

#### Identification of MDE metabolites

**Figure 4:**
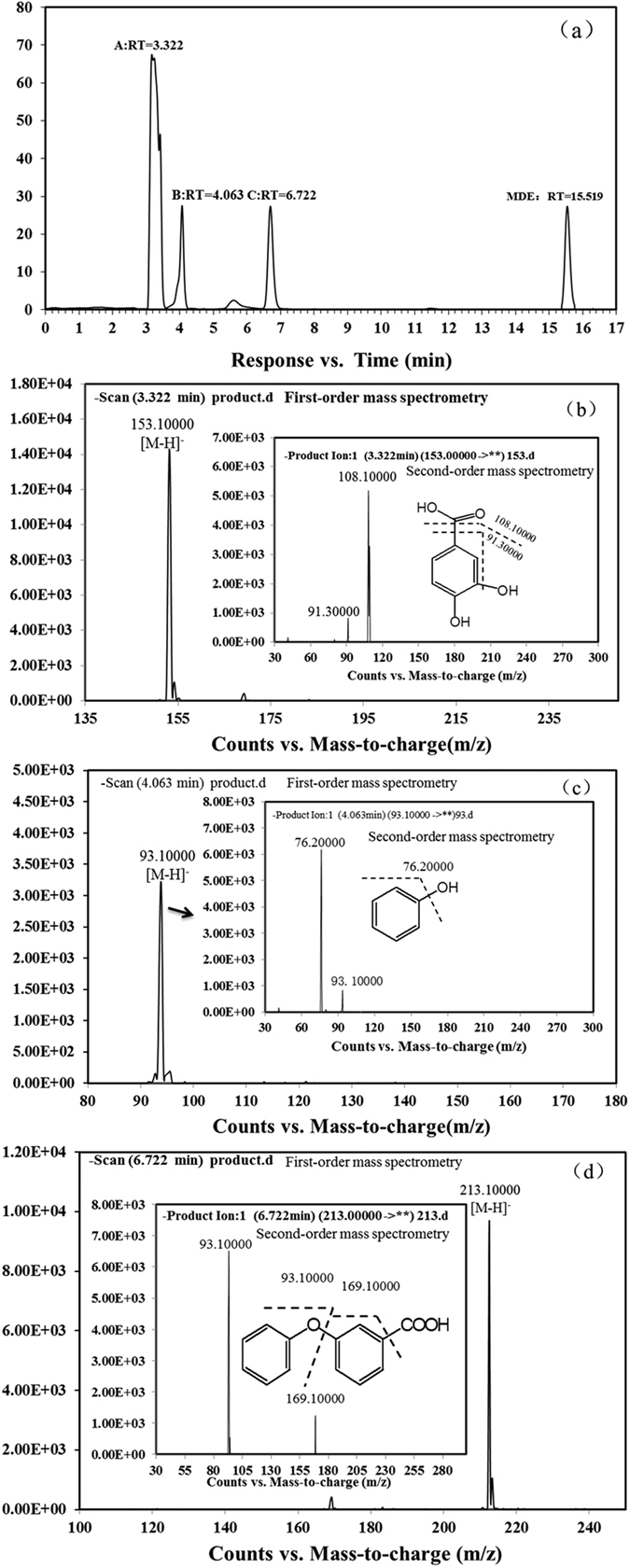
HPLC and MS/MS analyses of MDE transformation by strain QY7-2. (**a**). HPLC spectrum of metabolites generated during MDE degradation by QY7-2. (**b**) Tandem mass spectrometry of metabolite A. (**c**) Tandem mass spectrometry of metabolite B. (**d**) Tandem mass spectrometry of metabolite C.

During MDE degradation, three metabolites with retention times of 3.322, 4.063 and 6.722 min were analysed by HPLC-MS/MS (Fig. 4a). Metabolite A had a retention time of 3.322 min, which was equal to that of the authentic protocatechuate standard. Tandem mass spectrometry analysis showed a prominent protonated molecular ion at m/z 153.10000 [M-H]^−^ and characteristic fragment ions at m/z 108.10000 [M-COOH-H]^−^ and m/z 91.30000 [M-COOH-OH-H]^−^ (Fig. 4b). This tandem mass spectrometry feature is consistent with that of the protocatechuate standard. Thus metabolite A was identified as protocatechuate. Metabolite B had a retention time of 4.063 min, which was equal to that of the authentic phenol standard. Tandem mass spectrometry analysis showed a prominent protonated molecular ion at m/z at m/z 93.10000 [M-H]^−^ and a characteristic fragment ion at m/z 76.20000 [M-OH-H]^−^ (Fig. 4c). This tandem mass spectrometry feature is consistent with that of the phenol standard. Thus metabolite B was identified as phenol. Metabolite C, with a retention time of 6.722 min, which was equal to that of the authentic 3-phenoxybenzoic acid standard. Tandem mass spectrometry analysis showed a prominent protonated molecular ion at m/z 213.10000 [M-H]^−^ and characteristic fragment ions at m/z 169.10000 [M-COOH-H]^−^ and 93.10000 [M-(C_6_H_5_-COOH)-H]^−^ (Fig. 4d). This tandem mass spectrometry feature is consistent with that of the 3-phenoxybenzoic acid standard. Thus metabolite C was identified as 3-phenoxybenzoic acid. The HPLC spectra of the authentic standard chemicals were showed in Fig. S2.

**Figure 5:**
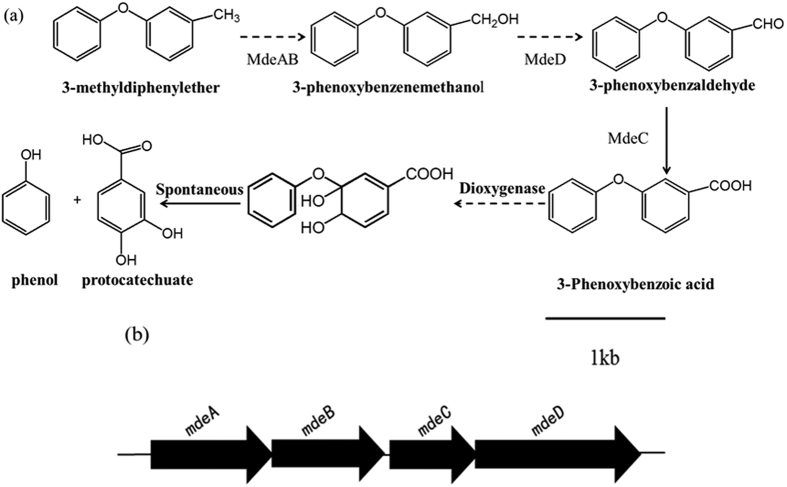
(**a**) Proposed MDE biodegradation pathway by strain QY7-2. The arrows in dash line represent hypothetical metabolites, the arrows in solid line represent detected metabolites (**b**) Physical map of the 4,805-bp gene fragments containing *mdeABCD* in strain QY7-2. The arrows indicate the size and direction of transcription of genes.

Based on the metabolite analysis, the biodegradation pathway of MDE by strain QY7-2 is proposed in Fig. 5(a). MDE was first metabolized by the oxidation of the methy on side chain to yield 3-phenoxybenzoic acid, which was then split into protocatechuate and phenol under the catalysis of a dioxygenase.

#### Cloning and sequence analysis of the *mdeABCD*

When QY7-2 is grown on MSM agar supplemented with 100 mg L^−1^ MDE, a transparent halo forms around the colonies due to that the unsoluble MDE was transformed to soluble metabolite. Therefore, inactivation of the initial methy-oxidation step halts the degradation process and consequently prevents the formation of the transparent halo. Accordingly, transposon mutagenesis was used to obtain mutants that failed to exhibit transparent rings around their colonies on LB plates supplemented with antibiotics and MDE. After the mutants were further screened by HPLC analysis for the loss of their capacity to degrade MDE, SEFA-PCR was used to isolate the genome DNA sequences flanking the Tn*5* insertion. Three mutants were obtained from approximately 6,000 transconjugants. Sequence analysis showed that the Tn*5* insertion sites of the three mutants were in the same gene, which indicated high similarity to a methy-oxidation gene, and the Tn*5* insertion sites of the two mutants were in the same position. Thus, we designated the obtained mutants as QY7-2-1 and QY7-2-2, and we chose mutant QY7-2-1 for further research.

A 4,805-bp fragment around the Tn*5* insertion in the QY7-2-1 mutant was amplified by SEFA-PCR; the complete physical map of this fragment is presented in Fig. 5(b). A computational analysis of this genomic region identified four complete ORFs, designated *mdeA, mdeB, mdeC* and *mdeD.* The protein sequences encoded by these ORFs were used as queries in a BLASTP search, and the functions were proposed for each ORF (Table 1). There is a 4-bp overlap between *mdeA* and *mdeB*. The deduced amino acid sequences of *mdeA* and *mdeB* were composed of 348 and 318 amino acid residues, respectively, and they exhibited high sequence homology to the genes encoding the toluene-4-sulfonate monooxygenase system iron-sulphur subunit TsaM (99%) and the reductase subunit TsaB (97%). The *mdeC* gene is located downstream (20 bp) of *mdeB,* and there is a 4-bp overlap between *mdeC* and *mdeD. mdeC* and *mdeD* exhibited high sequence homology to the genes encoding 4-formylbenzenesulfonate dehydrogenase TsaD (99%) and 4-(hydroxymethyl) benzenesulfonate dehydrogenase TsaC (99%). According to a previous report^14^, the *TsaMBCD* gene cluster encodes the enzymes that catalyse methy-oxidation; thus, it can be speculated that the *mdeABCD* gene likely encodes the enzymes catalysing the oxidation of the sidechain methy of MDE to form 3-PBA.

#### Functional expression of *mdeABCD* in *E. coli*

To identify the function of MdeAB, MdeC and MdeD, plasmids pETA, pETB, pETC and pETD, which contained *mdeA, mdeB, mdeC* and *mdeD,* respectively, under the control of a T7 promoter in the vector pET-29a(+), were introduced into *E. coli* BL21 (DE3). Then, the recombinant enzymes MdeA, MdeB, MdeC and MdeD, expressed in *E. coli* BL21 (DE3), were purified using Ni-nitrilotriacetic acid affinity chromatography. The purified enzymes gave single bands on an SDS–polyacrylamide gel (Fig. S3). The molecular masses of the denatured enzymes were approximately 38, 35, 29 and 53 kDa, which were in good agreement with the molecular masses deduced from the amino acid sequences.

**Figure 6:**
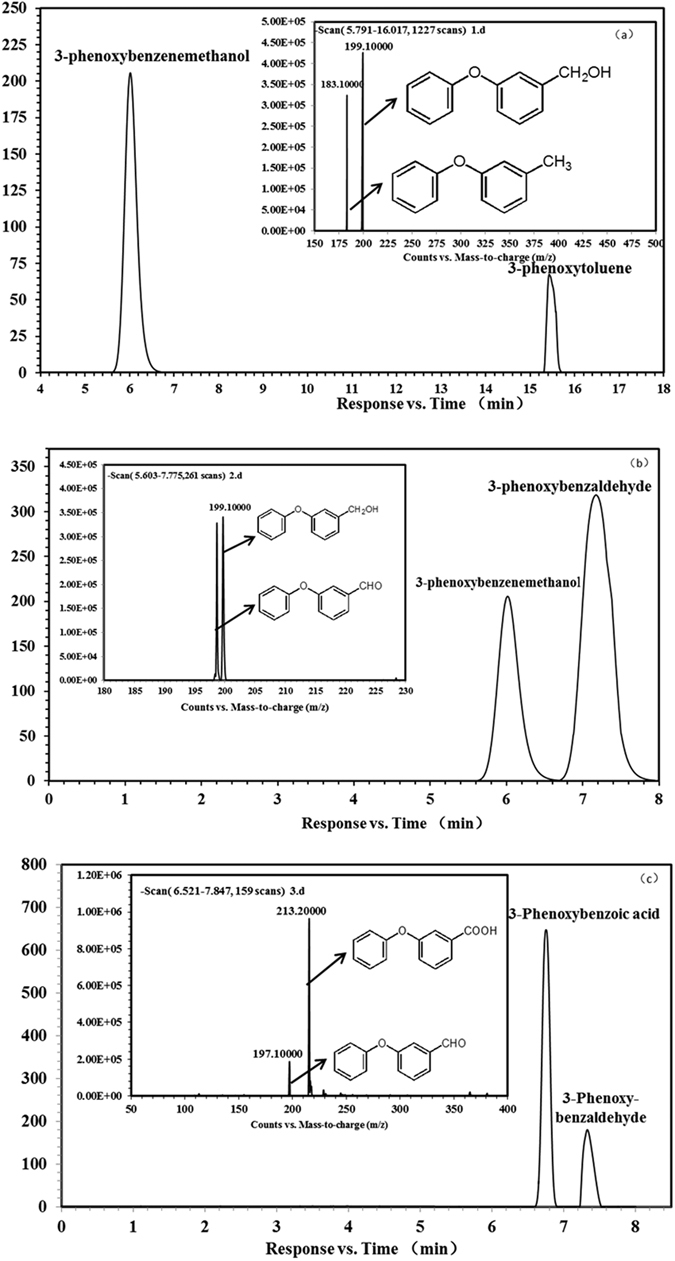
Chromatograms of the HPLC and LC-MS/MS catalytic effects by recombinant MdeAB (**a**), MdeD (**b**) and MdeC (**c**).

According to the spiking method with the standard compounds, the HPLC-MS/MS results demonstrated that MdeAB catalyses the transformation of 3-phenoxytoluene to generate 3-phenoxybenzenemethanol, MdeD catalyses the transformation from 3-phenoxybenzenemethanol to 3-phenoxybenzaldehyde, and MdeC catalyses the transformation 3-phenoxybenzaldehyde to 3-phenoxybenzoic acid (Fig. 6).

The enzymatic characteristics of MdeAB, MdeD and MdeC are shown in Table 2. The specific activities of MdeAB, MdeD and MdeC were 113.8 ± 3.5, 673.4 ± 8.7 and 274.5 ± 6.2 nmol min^−1^ mg^−1^, respectively. The optimum temperatures for MdeAB and MdeD was 35 °C, while the optimum temperature for MdeC was 30 °C. The optimum pH values for MdeAB, MdeD and MdeC were 7.0, 7.5 and 9.0, respectively.

## Materials and Methods

### Chemicals and media

The activated sludge used for bacterial isolation was collected from a wastewater treatment station in a pesticide factory located in the Jiangsu province in China. MDE (99.5% purity) was purchased from Sinopharm Chemical Reagent Co. Ltd. (Shanghai, China). Chromatographic grade methanol and acetonitrile were purchased from Sigma-Aldrich (St. Louis, MO, USA). All of the other reagents used in this study were of Analytical Reagent (AR) grade.

Luria-Bertani (LB) and mineral salts medium (MSM) were prepared as described in our previous work^15^,^16^. For preparation of the solid medium, agar powder was added to reach a concentration of 2.0%. The ultra-pure water used for the media was sterilized (autoclaved for 20 min at 121 °C) in advance. The MDE standard stock solutions (20 g^ ^L^−1^) were prepared in methanol and sterilized by membrane filtration (0.22 μm). The stock solution was sometimes added into the media according to the experimental design.

### Enzymes, strains and plasmids

The bacterial strains and plasmids used in this study are listed in Table 3. The *E. coli* strains were grown at 37 °C in LB broth or on LB agar. Other bacterial strains were grown aerobically at 30 °C in LB broth or on LB agar. The following antibiotics were used at the indicated concentrations: ampicillin (Ap), 100 μg/mL; spectinomycin (Sm), 100 μg/mL; gentamicin (Gm), 20 μg/mL; and kanamycin (Km), 50 μg/mL.

All of the enzymes used in the DNA manipulations were obtained from TaKaRa Biotechnology Co. Ltd (Dalian, China). *E. coli* DH5α and BL21 (DE3) were purchased from TaKaRa and were used for cloning and expression hosts, accordingly. The plasmids pMD19-T (TaKaRa) and pET-29a (+) (TaKaRa) were used as cloning and expression vectors, respectively.

### Isolation and identification of an MDE-degrading strain

Five millilitres of activated sludge was added into 100 mL of MSM (containing 100 mg^ ^L^−1^ MDE) to initiate the isolation process. The mixture was incubated on a rotary shaker (160 rpm at 30 °C) for 5 d. After that, an equivalent volume of supernatant (5 mL) was sub-cultured into the same MSM with MDE and was incubated. The whole procedure was repeated five times to achieve the enrichment step. Finally, the enriched cultures were diluted and spread onto solid MSM with MDE (100 mg L^−1^). A colony producing a visible transparent halo due to MDE degradation was selected and purified. As a control, medium without inoculation was maintained according to the same conditions. High-Performance Liquid Chromatography (HPLC) was used to detect the concentration of MDE. The isolate was identified according to morphological, physiological, and biochemical properties^17^ and 16S rDNA gene sequence analysis^18^.

### Degradation of MDE by QY7-2

#### Degradation characteristics of strain QY7-2

The isolated strain was pre-cultured in LB medium to achieve an optical density (OD_600_) of 1.0. Afterwards, the cells were collected (centrifuge at 8000 g for 3 min), washed with sterilized MSM, and resuspended in an equivalent volume of MSM. The inactivated cells were sterilized by autoclaving at 121 °C for 20 min. For all of the degradation assays, 2 mL of cell suspension was inoculated into 100 mL of MSM with 100 mg L^−1^ MDE, and the mixture was incubated in a rotary shaker (30 °C, 160 rpm) unless otherwise stated. As a control, medium inoculated with inactivated cells was maintained according to the same conditions. The degradation behaviours, under different temperatures (20, 25, 30, 35, and 40 °C) and pHs (4.0, 5.0, 6.0, 7.0, 8.0, 9.0, and 10.0), were investigated.

#### Kinetic study

The Monod model (Eq. (2)) was adopted to describe the degradation kinetics in this study.


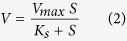


This equation was then transferred into the following:


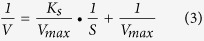


Where, *V* (h^−1^) and *V*_*max*_ (h^−1^) are the specific and maximum removal rates of MDE, respectively; *S* (mg L^−1^) is the final concentration of MDE; and *K*_*s*_ (mg L^−1^) is the half-saturation constant. The details of the conditions throughout the kinetic study were as follows: the initial concentration of MDE was 100 mg L^−1^; the inoculation amount was 100%; the temperature was 30 °C; the pH was 7 and the sampling time interval was 8 h. The MDE concentration of obtained samples were detected by HPLC to make a time-concentration curve. The slope of each point on the curve was obtained by Origin 8.0, which represented the degradation rate. The *V*_*max*_ and *K*_*s*_ were obtained by plotting *1/V* and *1/S*, where 1/*V*_*max*_ was the intercept and the *K*_*s*_/*V*_*max*_ was the slope.

#### Tn*5* mutagenesis and genome walking

The conjugation for the Tn*5* transposon mutagenesis with pSC123^19^ was performed on 0.45-μm pore size sterile membranes (diameter, 25 mm) placed on LB agar at 30 °C with a 1:1:1 donor-to-recipient-to-helper ratio. The Tn*5* transconjugants were selected on an LB plate containing 100 mg L^−1^ MDE and supplemented with Sm and Km. The mutants that could form a colony but failed to form a transparent halo around their colonies on the LB plates were screened by HPLC using a cell suspension assay as described above. The DNA flanking the Tn*5* insertion in the mutant was isolated using a genome walking method and a self-formed adaptor PCR (SEFA-PCR), as developed by Wang, *et al*.^20^. The 4,805-bp fragment was amplified from *Hydrogenophaga atypical* strain QY7-2 using the primer pairs listed in Table S1.

#### DNA manipulation, sequencing, and analysis

The isolation and manipulation of the recombinant DNA were performed as described by Sambrook and Russel^21^. The restriction enzymes, the PrimeSTAR HS DNA polymerase, and the TA cloning vector used in this work were commercial preparations and were used as specified by the supplier, TaKaRa Biotechnology Co., Ltd. (Dalian, China). The standard PCR mixture contained 10 ng of template DNA, 0.2 μM of each primer, 200 μM of each deoxynucleoside triphosphate (dNTP), 10 μL of 5 X PrimeSTAR buffer (Mg^2+^ Plus), and 1.25 U of PrimeSTAR HS DNA polymerase in a total volume of 50 μl. The standard PCR conditions were as follows: 98 °C for 30 s; 30 cycles of 98 °C for 10 s, 58 °C for 10 s, and 72 °C for 1 min kb^−1^; and 72 °C for 5 min. Oligonucleotide synthesis and DNA sequencing were performed by Invitrogen Technology Co., Ltd. (Shanghai, China).

BLASTN and BLASTP were used for the nucleotide sequence and deduced amino acid identity searches, respectively. For phylogenetic analysis, Clustal W was used to align all of the protein sequences^22^. The multiple sequence alignment was then imported into MEGA version 5.0 software^18^. A phylogenetic tree was then constructed using the neighbour-joining method. Distances were calculated using the Kimura two-parameter distance model, and a bootstrap consensus tree, inferred from 1,000 replicates, was taken to represent the evolutionary history of the sequences analysed. Predictions of the open reading frames (ORFs) were performed with ORF Finder (NCBI) and GeneMark^23^.

#### Expression of *mdeABCD* in *E. coli* BL21

The gene *mdeABCD* was amplified by PCR using Prime STAR HS DNA polymerase (Primers in Table S1) and was cloned into pET-29a (+) (kanamycin resistant) to obtain the primers in the constructs. The recombinant plasmids were then transformed into BL21 (DE3). The expression of these genes and the purification of the recombinant enzymes were implemented according to^24^ with slight modifications. Protein expression was examined via sodium dodecyl sulphate-polyacrylamide gel electrophoresis (SDS-PAGE).

The enzyme activity of MdeAB was determined by measuring the reduction rate of MDE. The optimized reaction mixture contained (in 0.5 mL) 25 μmol of potassium phosphate buffer (pH 7.0), 200 nmol of NADH, 40 nmol of FeSO_4_, and 50 μg oxygenase (MdeA) and reductase (MdeB) proteins. The enzyme activity levels of MdeD and MdeC were investigated by measuring the reduction rates of 3-phenoxybenzenemethanol and 3-phenoxybenzaldehyde as the substrate. The reaction mixture contained (in 0.5 mL) 25 μmol Tris/HCl, pH 7.0, 300 nmol NADH, 150 nmol of the substrate, and 100 μg of protein. All of the experiments were performed at 35 °C in triplicate. The control samples, without enzyme, were also analysed under the same conditions.

To investigate the activity of the enzyme under different pH values at 35 °C, the following buffers were used: 20 mM citrate buffer (pH: 5.0–6.0); 20 mM PBS (pH: 6.0–8.0); 20 mM Tris-HCl buffer (pH: 7.5–8.5); and 50 mM glycine-NaOH buffer (pH: 8.5–10.5). The optimal reaction temperatures were determined using the optimal pH at temperatures from 15 to 70 °C.

#### Analysis method

The optical density of the cell suspension was detected at 600 nm (OD_600_) using a UV spectrometer (PHILES D8). Colony-forming units (cfu) referred to the total number of bacterial colonies per unit volume and measured by diluting bacterial suspension onto LB plate and counting the number of bacterial colonies. The concentration of MDE was determined by HPLC with a reverse-phase C18 column. All of the liquid samples were filtered through a microporous membrane (0.22 μm) prior to the HPLC analysis. The mobile phase consisted of methanol, water, and formic acid (85:15:0.1, v-v:v), and the flow rate was 0.8 mL^ ^min^−1^. The wavelength was 230 nm, and the injection volume was 20 μL. A liquid chromatography/electron spray ionization tandem mass spectrometry (LC/ESI-MS/MS) analysis was performed to identify the metabolites using an Agilent G6410B triple quad mass spectrometer equipped with an HPLC (Agilent Technologies 1200 Series). The mass spectrometer was operated in negative-ion ESI mode. Other ESI conditions were as follows: gas temperature 350 °C, capillary voltage 4.0 kV, nebulization pressure 30.0 psi and gas flow rate 10.0 L min^−1^. The MS/MS conditions were as follows: fragmentor voltage 90 V and collision energy 15–20 eV.

#### Nucleotide sequence accession number

The nucleotide sequences of the 16S rDNA, *mdeA, mdeB, mdeC* and *mdeD* in *Hydrogenophaga atypical* strain QY7-2 had been deposited in the GenBank database under the accession numbers KX290851, KX290852, KX290853, KX809606 and KX809607 respectively.

## Conclusion

In the present study, the efficient 3-methyldiphenylether-degrading strain *Hydrogenophaga atypical* QY7-2 was isolated and characterized. Several metabolites of MDE were identified, and a new biodegradation pathway was discovered. The gene cluster encoding an enzyme system capable of MDE methy-oxidation was cloned and designated as *MdeABCD*. The recombinant enzymes MdeAB, MdeC and MdeD were expressed, purified and characterized. These data offer important knowledge on the fate of MDE in biodegradation and elucidate the biodegradation mechanism of MDE by strain QY7-2. Further research, such as the strain’s interaction with the environment, is still needed before strain QY7-2 can be applied in bioremediation.

## Additional Information

**How to cite this article:** Yang, Q. *et al*. Biodegradation of 3-methyldiphenylether (MDE) by *Hydrogenophaga atypical* strain QY7-2 and cloning of the methy-oxidation gene *mdeABCD. Sci. Rep.* **6**, 39270; doi: 10.1038/srep39270 (2016).

**Publisher's note:** Springer Nature remains neutral with regard to jurisdictional claims in published maps and institutional affiliations.

## Supplementary Material

Supplementary Information

## Figures and Tables

**Table 1:** Deduced function of each ORF identified within the 4805-bp DNA fragment.

Gene	Product size (amino acids)	Homologous protein	GenBank accession no.	Identity %	Proposed function
*mdeA*	348	Toluene-4-sulfonate monooxygenase system iron-sulfur subunit	P94679	99	monooxygenase oxygenase component
*mdeB*	318	toluene-4-sulfonate monooxygenase system reductase subunit	Q9AHG2	97	monooxygenase reductase component
*mdeC*	267	4-formylbenzenesulfonate dehydrogenase	P94681	99	dehydrogenase
*mdeD*	477	4-(hydroxymethyl) benzenesulfonate dehydrogenase	Q9AHG1	99	dehydrogenase

**Table 2:** Enzymatic characteristics of MdeABCD.

Enzyme	Specific activity (nmol min^−1^ mg^−1^)	Optimum temperature	Optimum pH
MdeAB	113.8 ± 3.5	35 °C	7.0
MdeD	673.4 ± 8.7	35 °C	7.5
MdeC	274.5 ± 6.2	30 °C	9.0

**Table 3:** Strains and plasmids used in this study.

Strain or plasmid	Characteristics	Source or reference
*Hydrogenophaga intermedia*		
QY7-2	Wild type; MDE-degrading strain; Sm^r^	This study
Tn*5* mutants	QY7-2; *mdeB*::*Tn5* Smr Kmr	This study
*E. coli* strains		
DH5α	*F*^*—*^*; recA1; endA1; thi-1; supE44; relA1; deoR; Δ (lacZYA-argF)U169;* Φ80dlacZΔM15	Takara
BL21(DE3)	*F*^*—*^; *ompT;* *hsdS_B_(r_B_^*—*^ m_B_^*—*^); dcm(DE3); gal; λ(DE3)*	Invitrogen
SM10_λ_pir	*thi thr leu tonA lacY supE recA* RP4-2-Tc::Mu λ*::pir*	Lab stock
HB101 (pRK2013)	Conjugation helper strain	Lab stock
Plasmids		
pSC123	Mariner transposon; Cm^r^ Km^r^	Lab stock
pMD19-T	TA clone vector; Ap^r^	TaKaRa
pET-29a(+)	Expression vector; Km^r^	Novagen
pETA	pET-29a(+) carrying *mdeA*; Km^r^	This study
pETB	pET-29a(+) carrying *mdeB*; Km^r^	This study
pETC	pET-29a(+) carrying *mdeC;* Km^r^	This study
pETD	pET-29a(+) carrying *mdeD*; Km^r^	This study
