# Supplementary material for: Biodegradation of 3-methyldiphenylether (MDE) by Hydrogenophaga atypical strain QY7-2 and cloning of the methy-oxidation gene mdeABCD
Source: Sci Rep. 2016 Dec 20;6:39270. doi: 10.1038/srep39270 (PMC5172442; doi:10.1038/srep39270)
Supplement: Supplementary Information [file srep39270-s1.pdf]

Biodegradation of 3-methyldiphenylether (MDE) by *Hydrogenophaga atypical* strain QY7-2 and cloning of the methy-oxidation gene *mdeABCD*

Qian Yang[a], Shu Cai[b], Shaowei Dong[a], Lulu Chen[a], Jifei Chen[a], Tianming Cai[a,*]

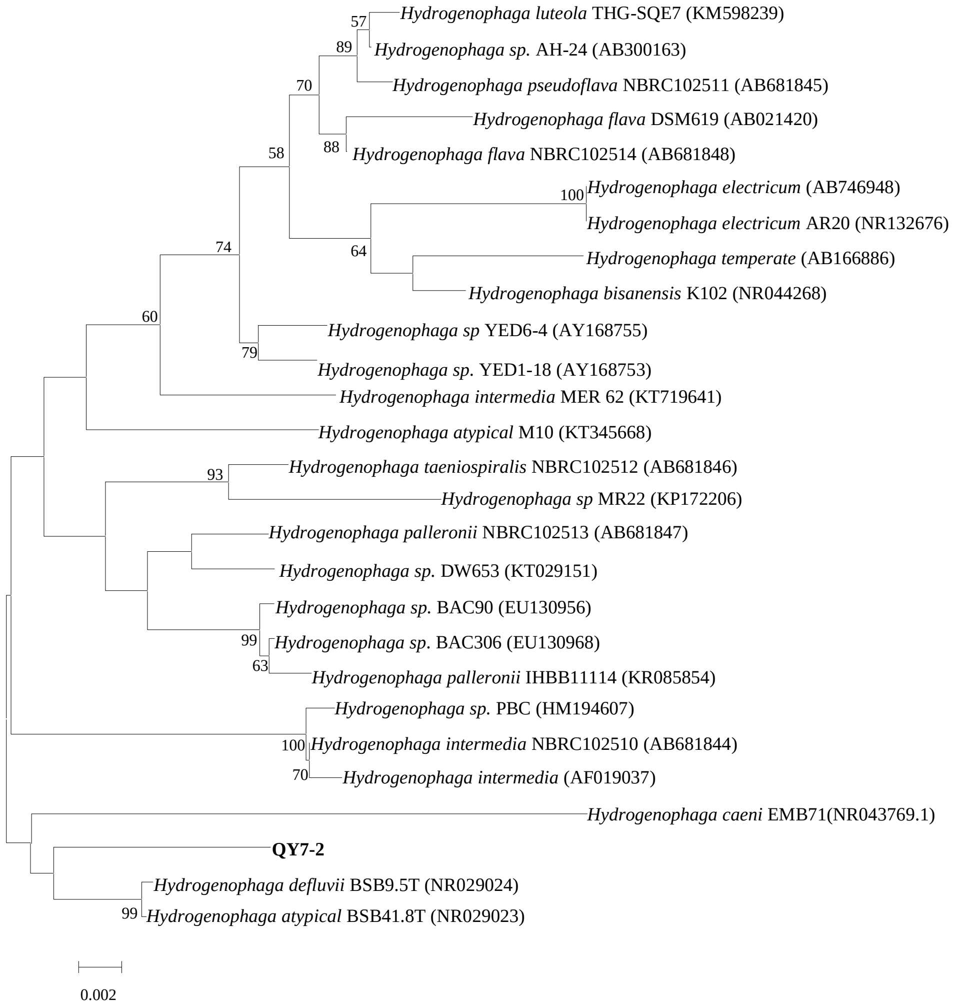

Fig. S1 Phylogenetic tree based on the 16S rRNA gene sequences of strain QY7-2 and related species. The GenBank accession number for each microorganism used in the analysis is shown after the species name. The scale bar indicates 0.002 substitutions per nucleotide position. Bootstrap values obtained with 1000 samplings are indicated as percentages at all branches.

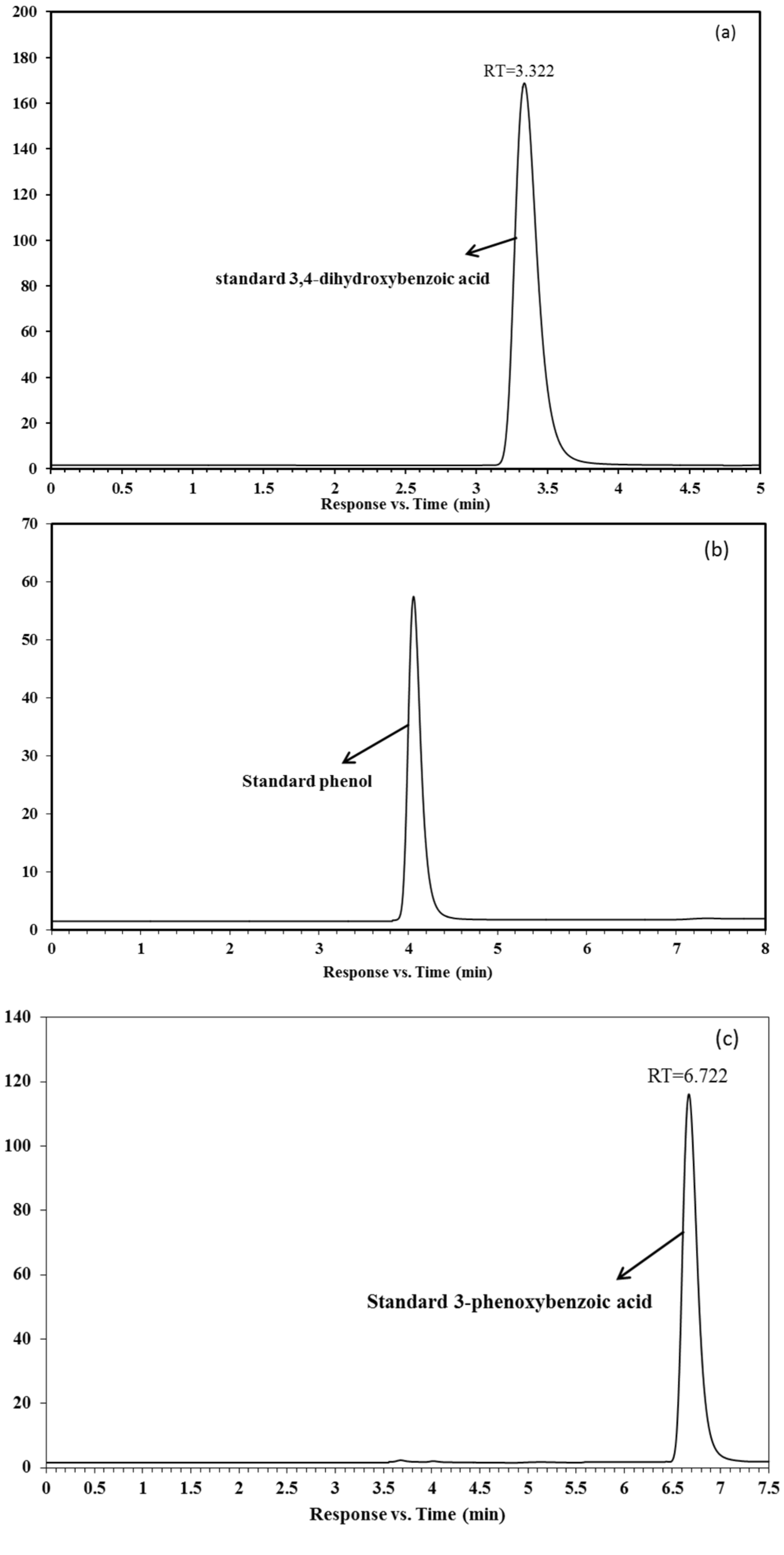

Fig S2 HPLC spectra of authentic chemicals. (a): 3,4-dihydroxybenzoic acid;(b)phenol;(c): 3-phenoxybenzoic acid.

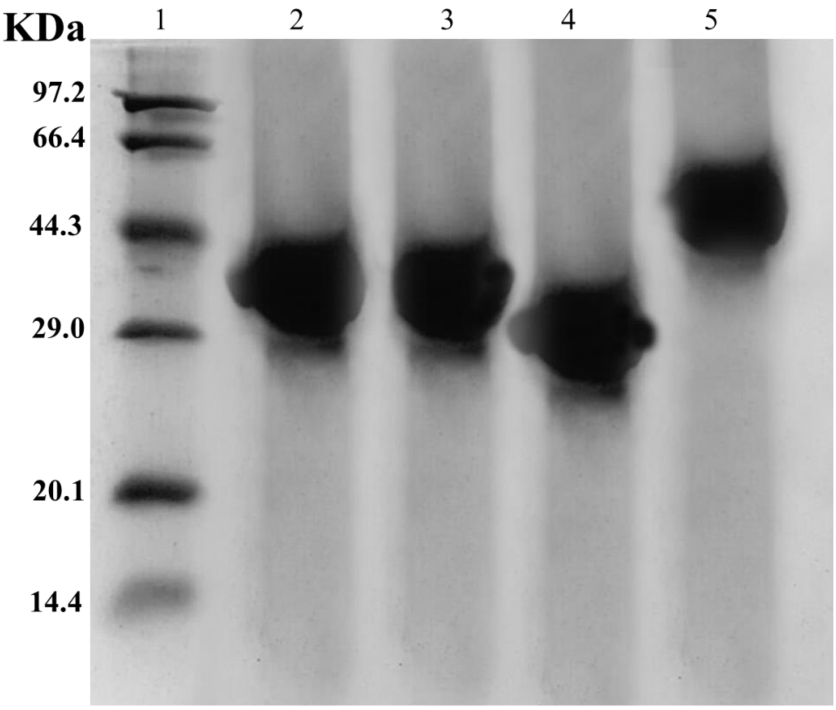

Fig S3 Analysis of the expression and purification of recombinant MdeAB,MdeC and MdeD.
Lane 1 protein molecular weight marker; lane 2 recombinant MdeA; lane 3 recombinant MdeB; lane 4 recombinant MdeC; lane 5 recombinant MdeD;

Table S1 Primers used in this study

Gene	Primers
***mdeA* F**	5'GGGAATTCCATATGTTCATCCGCAATTGCTGGTACGTGG3'
***mdeA* R**	5' CCGCTCGAGTAGAACGGCGGGACCGCGCCAG3'
***mdeB* F**	5'GGGAATTCCATATG AGCGCCGACGTGCCCGTGAC3'
***mdeB* R**	5' CCGACTCGAGTGATGTCCAGTACCAGCCGGCCG3'
***mdeC* F**	5' GGGAATTCCATATGAACCTGAACAATCAAGTGGCCATCG3'
***mdeC* R**	5' CCGCTCGAGTGTTGCGACCTCCGTCCACGT3'
***mdeD* F**	5' GGGAATTCCATATGAGCACCGTGCCCTACCGCT3'
***mdeD* R**	5' CCCAAGCTTGGGCCACGTAGTGCATGAACTT3'
**T5SP1**	5'TAACGGTTGTGGACAACAAGCCAGGGAT3'
**T5SP2**	5'GTAACGCACTGAGAAGCCCTTAGAGCCT3'
**T5SP3**	5'TACTAGCGACGCCANNNNNNNNNNCAGACC3'
